# Endamebic Pyorrhea and Its Complications

**Published:** 1915-02

**Authors:** J. S. Evans, William S. Middleton

**Affiliations:** Madison, Wis.


### ENDAMEBIC PYORRHEA AND ITS COMPLICATIONS.

A PRELIMINARY NOTE*

J. S. EVANS, M.D.

AND

WILLIAM S. MIDDLETON, M.D., MADISON, WIS.

The study of the relation of pyrrohea and oral foci of infection to systemic disorders of a probable toxic origin received a marked stimulus from the recent discovery of the causative relation of endamebae by Smith and Barrett,[1] 1914. In their study, the presence of the *Endamoeba gingivalis* Gros,[2] 1849, was constant in forty-six cases of pyorrhea. The independent works of Chiavero[3] and Bass and Johns[4] corroborate the constancy of this finding in gingival infections.

Prior to this investigation, the extensive clinical and experimental research in the subject of oral sepsis had shed considerable light on its relation as a distant focus for systemic toxemias. Hunter[5] had long advanced the theory of an oral septic origin for numerous systemic disturbances, more notably, pernicious anemia. More recently, Billings, Rosenow and others had emphasized the importance of oral sepsis as a factor in remote general conditions. The efficacy of autogenous bacterial vaccines had been tested, and the variability of results both locally and generally had opened the question of a possible complexity of causative factors.

---

* From the Department of Clinical Medicine, University of Wisconsin.

1 Barrett: Dental Cosmos, August, 1914; ibid., December, 1914.

2 The term "Endamoeba gengivalis" Gros, 1849, is substituted for "Endamoeba buccalis," Prowazek, 1904, at the suggestion of Dr. Allen J. Smith, whose studies of the original articles prove beyond a doubt the priority and accuracy of Gros' description and observations.

3 Chiavero, Angelo: Abstr., Dental Cosmos, September, 1914.

4 Bass and Johns: New Orleans Med. and Surg. Jour., 1914, lxvii, 456.

5 Hunter, Brit. Med. Jour., Nov. 19, 1904.

The application of the specific drug for the treatment of amebic dysentery, ipecacuanha or its active principle, emetin (alkaloid), to the treatment of an analogous condition in the mouth, was the next rapid step in our knowledge of pyorrhea. Two modes of procedure have been followed. The first, that of Smith and Barrett, was the local injection or application to the affected gums of a weak emetin hydrochlorid solution. At first a one per cent. solution of the hydrochlorid proved irritant in some cases, so that a strength of 0.5 per cent. has since been administered. The customary routine has been daily injections of a small portion, often only a fraction of a drop of this solution, into each pocket. Smith and Barrett,[6] in their preliminary report, noted the marked improvement to complete cure of thirteen cases of Rigg's disease so treated. Since that time many cases of correspondingly good results have been added to their list. Several ingenious devices have been evolved to meet the demands in certain cases referred by us to dentists. The use of air pressure to separate the gum from the tooth has been of assistance in some instances (Dr. S. H. Chase). The use of blunt-pointed needles (Dr. Kent Wood) and the replacement of the hypodermic needle by a dental file (Dr. E. J. Hart), the hanging drop of emetin solution adapting itself by capillarity to the smallest pockets where no needle could be introduced, have both been valuable refinements. The application of a heavy petrolatum to the gum margin after each treatment appears of value. Barrett, in a more recent article,[7] has carefully covered the ground of the surgical dental care, which must supplement any specific treatment.

The more recent announcement of Bass and Johns of their discovery of endamebae in eighty-five out of eighty-seven cases of Riggs' disease, and of its rapid subsidence

---

6 Barrett: Dental Cosmos, August, 1914.

7 Barrett: Dental Cosmos, December, 1914.

8 Smith, A. J.; Middleton, W. S., and Barrett, M. T.: The Tonsils as a Habitat of Oral Endamebas, The Journal A. M. A., Nov. 14, 1914, p. 1746.

after the subcutaneous administration of emetin hydrochlorid, constitutes the proof of the efficiency of a second method of treatment or of administration of the specific drug. Smith, Middleton, and Barrett,[8] in a recent study of tonsillar endamebae and their relation to systemic disturbances, noted in several cases the disappearance of endamebae from the cryptic contents on the subcutaneous administration of emetin hydrochlorid.

The present study constitutes a brief preliminary report of a series of cases under observation in the University of Wisconsin Medical Clinic. In all, seventy-two oral cases have been examined by the usual method, heated stage, warm slide and cover slip, the material scraped from the depth of the pocket being immediately mixed with warm normal saline and mounted. Seventy cases showed endamebae ranging in size from 8 to 32 microns in diameter, filled with granular material but no contractile vacuoles; the pseudopodia varied widely in size, length and activity; the ectoplasm was decidedly hyaline, and only after an interval was there an outpouring of the granular endoplasm into the newly formed hyalin pseudopod. These amebae fall under the heading of *Endamoeba gingivalis* Grcs. The type *Endamoeba kartulisi* Döflein, 1901, was associated with the *Endamoeba gingivalis* in two cases. According to the literature, the differentiation of these two types is to a great degree based on the difference in the size and activity of the pseudopods, those of the *kartulisi* being much broader and more active. Of these two points there could be no doubt in the cases noted; but an added differential point was observed, namely, the refractive quality of the substance of the psuedopods of the *kartulisi* was much more marked than that of the common forms. Indeed, the difference was almost that of hyaline and waxy renal urinary casts. An unclassified ameba was noted in two cases of pernicious anemia along with the type gengivalis. This ameba possessed a definite endoplasm and ectoplasm, was from 10 to 30 microns in diameter, its psuedopodia were rapidly thrown out, frequently subdividing, but never was there noted an outflow of the very highly refractile granular endoplasm into the pseudopodia. The two negative cases showed considerable gum retraction with dentine erosion.

A point to which our observation has led us to attach considerable importance is that of the mechanical interference by metallic filling and crown or plate attachments with the normal adaptation between the crusta petrosa or cement and the gum or pericementum (ligamentum circulare dentis of Kölliker). Invariably such cases have shown pyorrhea in these localities. Whether this is a constant finding and whether the presence of an irritating foreign body determines the condition, we can not judge at this time.

Of the seventy pyorrhea cases with amebae present, fifty-four were treated by the local injection of 0.5 per cent. emetin hydrochlorid solution. The results were practically uniform in the marked improvement of the pyorrhea. The period of treatment varied from four to twelve days, with an average of about seven. In a few instances the patients complained of "tender" gums, but the great majority remarked early in the course of their treatment on the new experience of an ability to brush their teeth without the pain or the bleeding noted prior to the treatment.

After an interval of from several days to a week following the last treatment, which corresponds to an apparent local cure, a re-examination for amebae was made. Even though the second examination was negative, as a precautionary measure, a subcutaneous course of emetin was instituted. Two plans of dosage were followed: a series of small doses daily, $\frac{1}{8}$ grain doses of the hydrochlorid for eight days or, a shorter series of larger doses, $\frac{1}{2}$ grain repeated with a day intervening followed by $\frac{1}{4}$ grain on two successive days. The reason for this supplementary treatment has been to insure the death of any endamebae penetrating the deeper tissues of the gums, and therefore possibly unscathed by the local injections. Another rational procedure would be periodic subcutaneous injections, say at monthly intervals over several months, to prevent the multiplication of encysted amebae which resist the biochemical amebicidal action of ipecac.

Only five cases have been treated with subcutaneous injections alone. The results have been entirely satisfactory, but with ambulatory cases we have not felt justified in using the rather large doses suggested by Bass and Johns. To us, this very fact constitutes a real drawback to the use of the remedy subcutaneously alone.

The constitutional manifestations associated with endamebic interstitial gingivitis are the disturbances attributable to a toxemia of any other origin. Fifty-two, or 74 per cent. of the seventy pyorrhea cases showing endamebae, displayed constitutional symptoms or disturbances of more or less marked degree. We have endeavored to group the constitutional derangements in these cases attributable to a toxic factor under several heads: (*a*) arthritic, (*b*) neuritic, (*c*) digestive, (*d*) hemic, and (*e*) miscellaneous.

Under these headings twenty-six individuals, 50 per cent. of the persons exhibiting constitutional symptoms or 27 per cent. of the total endamebic, pyorrhea cases, showed arthritic symptoms of varying degrees, most frequently, however, of the type denoted as arthritis deformans. Six persons, 11 per cent. of the total constitutionally affected, consulted us for neuritic involvement, and a similar percentage showed hemic changes. Two patients suffered from obscure digestive disturbances. The remaining thirteen individuals falls into a miscellaneous group, whose components cover a wide range. Certain of these will be discussed individually. In such a broad general classification we have been forced to pick out the most prominent symptom of a toxic complex, and we have therefore sacrificed the complete picture for the purpose of presenting its most evident detail.

The application of a specific drug for the relief of a supposedly specific infection is an acknowledged therapeutic principle. The further application of this principle to the relief of remote conditions complicating the original specific lesion is justified. However, in this instance we are not compelled to assume an amebic character for the remote disturbance arising from the primary gingival focus. The mere relief of a focus of toxic absorption frequently explains the subsequent alleviation of a toxic derangement.

Seventeen of our twenty-six arthritic patients have received local treatment with marked improvement or the absolute relief of the interstitial gingivitis in all cases. Of these patients, ten have experienced decided improvement subjectively in their joint symptoms; three have had but slight relief from their arthritic pain, and the remaining four have shown no definite change. Two arthritic patients not treated locally, together with thirteen from the foregoing group undergoing local treatment (nine of these were improved and four unimproved under the local care), have been treated by the subcutaneous administration of emetin hydrochlorid. Of the four patients unimproved by local treatment of the gingival condition, none has shown maintained improvement even under the combined treatment. Two of these to the roentgenogram show well-marked osteo-arthritis. A third is undoubtedly a case of traumatic bursitis, in which the arthritis is a secondary factor, and the fourth is a very extreme long-standing case of arthritis deformans. In all, nineteen cases of arthritis have received the various forms of treatment with no apparent improvement in four cases, with indifferent results in three, but with decided unmistakable improvement in twelve cases, 63 per cent. of those treated, two of these being treated by the subcutaneous method alone.

The group of neuritis cases has been disappointing because of our inability to follow the subsequent course of five of the six persons referred to dentists for local care.

The only patient returning for the supplementary subcutaneous treatment has shown no change in the neuritic condition.

Of the two cases with digestive derangement as the prominent symptom complex, both were improved by local treatment, further improvement being noted in one following the administration of emetin hydrochlorid subcutaneously. In both cases malnutrition was the most important factor, though neuroses usually attributable to general toxic disturbances were manifest. We believe that the improvement in these cases was due partly to the removal of a toxic focus, thereby correcting a metabolic disturbance, and partly to the betterment of the local condition in the mouth, which was followed by improved mastication and increased appetite.

The six cases with hemic disturbances included two cases of pernicious anemia, and four simple secondary anemias of rather moderate grade. We have been unfortunate in our inablity to follow the pernicious anemias. One of them was started on emetin subcutaneously, but a return of a previously existing gastro-intestinal disturbance made its continuance inadvisable. A gain of up to 500,000 in the total red corpuscle count was noted in several of the secondary anemias on the removal of the gingival toxic focus by the emetin treatment. Similar changes in the blood picture of several cases grouped elsewhere have been noted after the subsidence of the pyorrhea.

We freely admit a question of doubt as to the etiologic relation in the group of miscellaneous conditions associated with pyorrhea. However, the relief of certain remote symptoms and conditions, which undoubtedly had a toxic focus as a basis, under the emetin treatment has led us to include the whole number. For instance, a case of obstinate uncontrollable headaches, whose source could not be discovered, responded quickly on the relief of a pyorrhea. A still more obscure case of marked splenic enlargement or tumor without a blood picture characteristic of any pathologic lesion was observed for two months without a diagnosis and without any change in size. There was, however, a most extreme pyorrhea. The local improvement in this condition under the local use of emetin hydrochlorid was attended by a reduction of the splenic tumor of 1½ inches in both the longitudinal and transverse diameters; a total reduction of over 2 inches in both diameters has followed the combined local and subcutaneous administration of the same drug. Other associated conditions noted in this miscellaneous group include accessory nasal sinusitis, endocarditis, continued unexplained leukocytosis, and the toxic cases falling under a broad, general heading of lowered nutrition and lowered general vitality. Among this type of cases only continued treatment and observation can possibly note a change of condition. Additional cases of the nature of visceral parenchymatous degeneration, as occur in the myocardium, liver and kidneys in any toxemia, will possibly be noted in the pursuance of this study.

The question of local reaction on the subcutaneous injection of emetin hydrochlorid in our cases has been most interesting. For instance, a case of arthritis whose pyorrhea has been cleared up by local treatment will show no reaction at the points of subcutaneous injection of two successive half-grain doses of emetin. On the third or fourth dose of only one-fourth grain, a most marked local reaction in the form of a wide, indurated areola of inflammation about the point of injection occurs. This local reaction may be attended by a temporary exacerbation of the general symptoms, usually followed by a marked improvement. We have been led to think of this reaction as an index of a point of saturation, when, with the death of numerous amebae the strength of emetin in the blood having reached a lethal point, a great amount of bacterial and possibly amebic toxin is liberated. Of course, this explanation is tentative, and subsequent observations may rule it out, but the alleviation of the general symptoms succeeding such an occurrence strongly supports this view.

As to the manner of the production of the toxic reaction in structures remote from the gingival focus, the several explanations advanced by Smith, Middleton and Barrett[8] might be quoted:

> Much must depend on the local circumstances governing absorption, and much, too, must depend on the number and type of associated bacteria. Whatever other relation may obtain between the amebas and the accompanying vegetable micro-organisms, one may be certain that the amebas feed largely on the latter; and in this bacterial phagocytic action they doubtless set free from this and from that organism different endotoxins. Doubtless bacterial toxins thus originating play a more important part than do toxins from the amebas themselves, if there be amebic toxins.

In regard to the exact method of action of either toxin, we may assume the selective action of the specific toxins to determine the system or the structure involved. In this connection, the old theory of the metabolic origin of many of the complications mentioned above may present itself in a new light. The liver is to a great degree responsible for the disposal, breaking up or removal of toxins arising within the body. It is, therefore, barely possible that certain of the systemic disorders may arise from a disturbance of the normal metabolic function of the liver. As to the hemic complications, the degrees of secondary anemia noted might readily arise from a bacterial hemolytic toxin, but our observations on the active lysis of engulfed red corpuscles in numerous instances lead us to the belief that the endamebae either through an intracellular or extracellular hemolytic toxin, or both, may be even a more important factor.

In conclusion, we would impress the facts that constitutional disturbances are very frequent complications of endamebic pyorrhea, that arthritis, particularly of the type arthritis deformans, is the most frequent complicating disorder, and that the results from the local and general administration of emetin hydrochlorid in the relief of the pyorrhea and the marked improvement of the arthritic conditions are very encouraging in a large percentage of cases. In addition, unexpected relations between pyorrhea and certain remote conditions are established through the response to the emetin treatment.

We are indebted to the clinical staff of the University of Wisconsin, to the dentists of Madison and to Dr. Allen J. Smith of the University of Pennsylvania for assistance in the treatment of this series of cases and for timely suggestions during this period of preliminary observation.

*Journal A. M. A.*, January 30, 1915.

